# Industrial policies of integrated energy services in China: A perspective of qualitative analysis

**DOI:** 10.1016/j.heliyon.2023.e22360

**Published:** 2023-11-14

**Authors:** Shengzhong Huang, Can Zhang, Wei Li, Mengkai Liu

**Affiliations:** a School of Management, China University of Mining and Technology -Beijing, Beijing, 100083, China; b Research Institute of Decision-making Science and Big Data, China University of Mining and Technology -Beijing, Beijing, 100083, China

**Keywords:** Integrated energy services, Energy restructuring, Industrial policy, Government intervention, Qualitative analysis research

## Abstract

Integrated energy services (IESs) are a systematic improvement and structural optimization of energy from production to consumption. However, many studies on IESs have only focused on typical cases or engineering technologies. In this study, we adopted a qualitative analysis research method to qualitatively analyze 21 important policy documents related to IESs in China from 2015 to 2022. The research provides an overview of the policy landscape and the key incentives for IESs implementation in China, identifies the challenges and opportunities for further expansion of IESs beyond industrial parks, then discuss the implications for future research and policy development. The research of the policy documents reveals that IESs as a means to improve energy efficiency and reduce carbon emissions, provides a deeper understanding of the potential of IESs in promoting energy security and mitigating climate change in China and the world, and strives to bridge the gap between IESs engineering sciences and social sciences.

## Highlights

• IESs are a central concept covering integrated energy solutions including efficiency, renewables, and demand management.
• The IESs require adaptation to the changing realities in the energy restructuring process.
• Understanding the relevant industrial policies of IESs is necessary.
• The externalities and public properties of IESs industrial policy are discussed.
• Use qualitative analysis research methods instead of pure technical point of view to study the IESs.

## 1 Introduction

Integrated energy services (IESs) prioritize renewable energy and integrate various electricity-based energy sources. IESs deeply integrate energy and information systems and can realize mutual transformation and optimal allocation of multiple energy sources. This provides users with innovative energy applications or services to achieve large-scale energy savings and consumption reduction and can effectively promote low-carbon green development of economy and society [1,2]. IESs were first introduced in October 2016 in the “Measures for the Management of Orderly Liberalization of Electricity Distribution Business,” an official public policy document in China. Power operators were then allowed to provide value-added services to customers for a fee, including various energy optimization combination programs that provide smart energy services (e.g., power generation, heating, cooling, gas supply, water supply). This denotes the germination of IESs in China. Since 2017, the State Grid Corporation of China (SGCC) has issued official documents formally announcing its transformation into a novel IESs provider, and several energy enterprises attempted to start an IESs business. Since then, IESs formally debuted on the stage of China's energy industry.

According to World Energy Statistics, although global energy demand and carbon emissions decreased by 4.5 % and 6.3 %, respectively, in 2020, global fossil energy demand grew by 5.8 % in 2021, constituting the largest increase in history [3]. With the global economy now recovering from the pandemic, combined with turbulent international conditions, prices and demand for natural gas are simultaneously rising worldwide, while coal consumption growth remains expected but worrying. Coal consumption and prices remain on the rise in the European Union (EU), and China and India accounted for 70 % of coal demand growth in 2021. This reveals that economic growth and energy security are more important than achieving “global temperature control of 1.5 °C,” and that the world remains in a carbon emissions growth cycle [3,4].

**Fig. 1:**
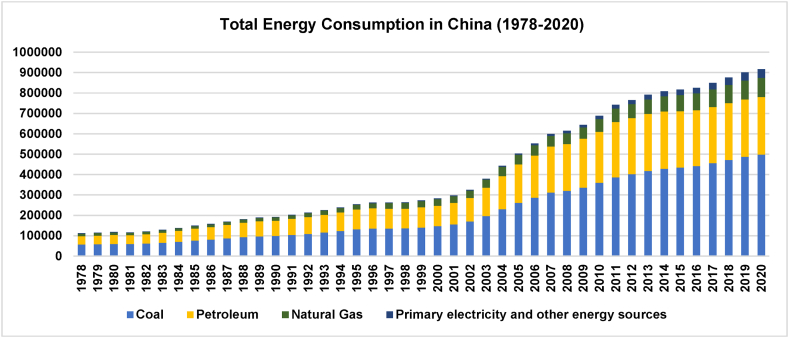
Total energy consumption in China (1978–2020).

For China, ensuring energy security is a key national task in the energy transition and adjustment process, and implementing IESs is particularly critical. After more than half of a century of construction, China has become the world's-largest energy production and consumption country. In energy utilization, China has a well-established production and supply system of coal, oil, natural gas, electricity, nuclear power, and new and renewable energies [5,6]. Fig. 1 indicates that the total annual coal consumption remained strong from 1978 to 2020, despite shares of coal in China's total energy consumption decreasing yearly. However, responding to the 2015 Paris Agreement to mitigate climate change, huge pressure on carbon emissions has led China to propose an emission peak and carbon neutral target in 2020. China has ample room for policy implementation and a very extensive energy management toolbox. The IESs, one of China's vigorously pursued energy industry policies, can optimize the energy production, supply, distribution, and consumption system and reduce carbon dioxide emission from the energy sector.

Conversely, as in Fig. 1 above, although China's share of coal consumption is gradually decreasing, the total consumption of traditional fossil fuels in China continues to rise, and there is a lot of potential to reconfigure and improve China's energy structure. However, the IESs implementation still requires adaptation to these changing realities in the energy restructuring process. Hence, understanding the relevant industrial policies of IES is important. Therefore, this study adopts a qualitative analysis research (QAR) method to analyze 21 important policy documents related to IESs in China between 2015 and 2022 and, accordingly, discuss the current status and policy implications of China's IESs industrial. The contribution of this research mainly includes the following two aspects.

i) Compared with previous studies on IESs from the perspective of technology, this study uses the QAR method and focuses on IESs-related industrial policy documents to analyze the development trend and policy implications of IESs in China.
ii) Based on this study, key objects for implementing IESs can be more clearly defined (i.e., various industrial parks with concentrated energy consumption and high-carbon emissions). Additionally, we discuss the typical customers of IESs in industrial parks and further studies.

The remainder of the paper is organized as follows. Section 2 reviews literature about the externalities and public properties of IESs industrial policy. Section 3 introduces our data and discusses the results. Section 4 presents the energy policy implications of China's IESs. Section 5 is the conclusion.

## 2 Literature review

IESs are based on energy restructuring, and IESs implication requires balance among interlinked energy restructuring [7,8]. While IESs are closely linked with energy restructuring, the transformation or improvement of energy structure cannot be effectively regulated by the market economy alone and must rely on active government participation. Joint global efforts to maintain global warming increases within 1.5 °C illustrate that climate change and energy transformation are part of international global public governance (i.e., a public good) and cannot be tackled through market mechanisms [9,10]. Market regulation can be implemented at the microgovernance level, however, energy has significant public attributes and requires local and national government initiative and guidance. Owing to the strong externalities of public goods, a certain degree of market failure in energy production and supply is found. Government intervention through industrial policy can achieve economic and social goals and, hence, can be used to appropriately correct market failures [11].

Without proactive government intervention, environmental protection and low-carbon green development goals remain difficult. In order for the government to participate and intervene, it is essential to comprehend the government's intentions, which are frequently conveyed through the policies they formulate [12,13]. Therefore, it is crucial to understand these policies and the underlying forces behind them. In contrast to prior research in the field of engineering technology, this paper concentrates on industrial policy.

### 2.1 IESs and energy restructuring

Energy restructuring requires significant changes in the proportion of each energy production or consumption, resulting in changes in both subjective and objective factors. The former include energy policy adjustments and climate change, and the latter include energy depletion, energy costs, and supply shocks. The energy structure, which is a multilevel concept, combines energy, economic, social, cultural, and technological factors [14,15]. In order to implement IESs successfully, a range of energy engineering technologies is required. Prior to exploring the industrial policy of IESs, it is essential to gain a brief understanding of the development trends of IESs and energy restructuring, from the perspective of energy engineering technologies [[16], [17], [18]]. According to the understanding and practice of integrated energy and energy restructuring, IESs development should be adapted to future diversification of decentralized energy sources—clean technology and high efficiency and low carbon.

i) Type of energy consumption is shifting from centralized and single to decentralized and diversified. Most countries worldwide have gradually shifted from a single traditional fossil energy source to diversified energy sources, especially on the rapid growth in the share of renewable energy sources [14,19]. For example, in the US, until the 1970s and 1980s, energy sources mainly relied on coal, oil, and natural gas. After entering the millennium, wind power, hydropower, shale gas, shale oil, solar energy, geothermal, and other energy sources have accounted for an increasing share, and energy sources are becoming more diverse.
ii) Energy utilization technologies are shifting from highly polluting to more environmentally friendly. Technological advances in energy have facilitated gradual shifts from high-pollution and low-efficiency to clean and ecofriendly energy [14]. In the US, coal-fired power generation presented a steep decline after 2005, and natural gas-fired power generation began overtaking coal-fired power generation in 2015. Simultaneously, high technology shale gas and oil extraction and various clean energy utilization technologies are widely used. Nuclear and wind power are presenting a rapid upward trend, with nuclear power generation ranking third in the US in terms of power generation.
iii) Energy consumption is shifting from inefficient and high-carbon to efficient and low-carbon. To control total carbon emissions and curb the greenhouse effect, most countries worldwide have started promoting carbon emission reduction to manage climate change. Energy consumption is presenting a transition from high-carbon to low-carbon, with traditional fossil fuel consumption presenting a downward trend, and the proportion of low-carbon renewable energy and other clean energy consumption is increasing [20,21]. For example, in France, the share of renewable and nuclear energies has been increasing, with the former accounting for almost half of current total national energy consumption. Japan, owing the Fukushima nuclear meltdown in 2010, has seen a more significant decline in nuclear energy use.

### 2.2 Externalities in implementing the industrial policy of IESs

While numerous studies on IESs focus on the engineering challenges, examining the externalities, particularly the negative ones, that arise from the implementation of IESs can offer engineers a new perspective from a social science standpoint, which is equally valuable [18,22,23]. Externalities (i.e., external effects) refer to the positive or negative impact of economic activities on other economic agents. Positive and negative externalities exist, and the latter leads to loss of efficiency, which can also lead to market distortion or failure [24]. In neoclassical economic theory, the market mechanism resolves resource allocation. Its price mechanism will promote economic agents to allocate resources efficiently and achieve Pareto optimality. However, externalities violate inherent rules between marginal utility and market pricing under utility maximization, becoming an inefficient form of pricing. This, in turn, promotes inefficient resource use by economic agents seeking to maximize efficiency [25]. In summary, if externalities remain, market equilibrium stays efficient, and the externalities must be corrected to improve market efficiency.

From an economic perspective, the traditional energy consumption structure increases the negative externalities. This leads to overconsumption of inefficient and high-carbon energy, which will cause environmental pollution and damage. Negative externalities cause inconsistency between the private and social marginal costs, so there will be a loss of social welfare [26]. Energy restructuring can reduce environmental pollution, improve effective resource use, and increase opportunities for other people and future generations to use resources, which improves the long-term development and overall welfare level of society [25,27]. However, the scarcity of energy resources and public nature of environmental resources determine the public goods attribute of energy restructuring. This makes it difficult for the market price mechanism to allocate resources in the process of energy production and supply [26,28,29]. Additionally, as energy restructuring has positive externalities, relying solely on the market can lead to inadequate supply. Energy shifts to high efficiency and low carbon, and this transition is reflected in the externalization of social benefits and economic benefits. Hence, their private benefits are smaller than social benefits, and contemporary benefits are smaller than multigenerational benefits; hence, the private sector is undersupplied.

### 2.3 IESs may exhibit market failure due to their public goods properties

Public goods are goods that can be consumed without reducing the amount of that good for other consumers. Private goods are goods that can be divided and then distributed in parts to different individuals at competitive market prices, while having no externalities on other consumers. Public goods are characterized by ‘nonexcludable consumption’ and ‘nonrival consumption’ [30,31]. Both consist of the basic criteria for distinguishing public goods from private goods. Some economists refer to products that satisfy only one of the characteristics as quasipublic goods, which are mainly public goods but have characteristics of private goods [32]. Based on these arguments, it can be said that IESs possess certain characteristics of a public good. Therefore, exploring the industrial policy of IESs through the lens of social science research methods appears to be a interesting avenue [23,33].

In perfect competitive economies, the supply and demand for private goods are balanced based on the principle of marginal costs equal marginal benefits. However, the nonexcludable and nonrival consumption characteristics of public goods are inconsistent with the assumption of competitive economic efficiency. Such positive externalities create false incentives for consumers, while the incomplete transparency of market signals may lead to underprovision of public goods, making them dependent on others to provision public goods instead of providing them themselves [32,34]. Owing to the awareness, conditions, and economic rationality, markets have limitations in the provision of public goods. Furthermore, “free-riding” leads to an inadequate supply of public goods by private individuals. However, although market mechanisms may be introduced to allocate public goods, they achieve the principle of “marginal cost equals marginal benefit.” In this situation, the government must participate in providing public goods and play key roles in the market [35,36].

IESs development should be adapted to the trend of efficient and clean energy. Increasing the proportion of efficient and low-carbon energy consumption can rarely bring direct benefits; however, positive externalities will indirectly bring greater social and environmental benefits [4]. Clean renewable energy can change energy consumption structure, and the benefits of energy restructuring mainly bring positive effects to other market players (e.g., environmental improvement, energy savings, and sustainable social development). Hence, energy restructuring can have “spillover” benefits, wherein other market players and future generations do not need to pay energy restructuring implementers to enjoy the benefits [20,24]. However, if the government were to prevent these actions, it would be too costly or impossible. From this perspective, IESs and the benefits of energy restructuring are typically nonexcludable. Conversely, from a nonrival perspective, IESs have certain nonexcludable characteristics of quasipublic goods, with the risk of market failure. Discussions on the economics of IESs suggest that employing research methods from humanities and social sciences, such as QAR, may be more advantageous than relying solely on engineering and technology research methods [33].

### 2.4 IESs market necessitates government intervention using industrial policy instruments

Neoclassical economic theory suggests taxing associated with external costs and subsidizing associated with external benefits. Hence, social and private marginal costs remain equal, aiming to provide more efficient source allocation [11,37]. In essence, IESs are one of the directions of energy restructuring. Owing to the externalities of energy restructuring, which may cause energy market failure, market mechanisms alone cannot be expected to promote or reduce efficient supply of clean energy and of highly polluting energy, respectively. To achieve socially optimal resource allocation, governments could intervene through executive orders or industrial policies to weaken externalities and solve the dilemma of market failure [38,39].

When implementing IESs, the government also needs to combine market mechanisms (e.g., constraint or incentives policies) to improve market efficiency. Hence, when the government uses appropriate fiscal policies, as fiscal policies combine incentives and penalties, if well-coordinated with market mechanisms, this can effectively improve the government's supply of public goods [40]. Just as public infrastructure like highways—being public or quasipublic goods—are unlikely to be provided by the market, they remain as susceptible to “free-riding” effects. Thus, for public goods like highways, the government can intervene through policies (e.g., charging highway tolls) to stimulate the supply of highways and achieve its development goals [41].

Generally, IESs are ultimately related to energy restructuring, and the relevant policy instruments are fiscal and tax policy instruments. These mainly include special tax exemptions, deductions, credits, accelerated depreciation, capital expensing, preferential tax rates, and so on. In implementing new energy businesses, fiscal and tax policies have the most extensive incentive mechanism. Internationally, tax incentives are called government tax subsidies, and their incentive mechanism is to influence the cost-benefit of a specific target through fiscal policy instruments, indirectly influencing them to change their energy production or consumption [40,42,43].

i) Governments may provide support for investment, production, transportation, consumption, and sales through tax and subsidy incentives for specific types of energy sources or enterprises, including energy-consuming products that satisfy certain criteria.
ii) Governments may deploy specific oversight or controls for specific energy enterprises through regulatory incentives—particularly for new technologies or potentially hazardous facilities—to increase public confidence and acceptance. Nuclear power plants, for instance, are mandatorily regulated by the government to ensure public health and safety.
iii) Governments may use funds to support research and demonstration projects to encourage enterprises and research organizations through R&D and investment incentives to conduct energy projects encouraged by policy, promote the energy structure toward to the desired goals, or direct market investment in certain types of energy sources and technologies.
iv) Governments may mandate the procurement of specific energy products or services to reduce costs or expand market scale through government procurement. In China, government procurement is often combined with energy product certification and energy efficiency labeling systems, such as the “Government Procurement List of New Energy Products” promulgated by government.

Traditional fossil energy sources have negative environmental impacts, and while various policies can solve this externality, economists tend to use policies (e.g., emissions taxes) to address this externality, owing to significant efficiency advantages [4,29]. Currently, with regard to controlling greenhouse gas emissions, some countries adopt policies including cap-and-trade, while policy discussions in other countries have shifted to imposing carbon taxes or levies on carbon emissions. Additionally, to effectively reduce fossil fuel consumption, low-carbon alternative energy can be subsidized; however, this may not be economically efficient [44,45]. Therefore, although policy incentives are intended to improve economic efficiency, if subsidies are derived from taxes, which cause market distortions, the policy may be inefficient and fail to achieve the purpose of the incentive. To effectively utilize policy tools for industrial guidance, it's essential to comprehend the government's policy interventions [17,23,33]. Thus, when implementing IESs, it's important to clarify their key objects and typical customers. This is one of the primary motivations behind this study, which aims to interpret and analyze the industrial policies of IESs using the QAR method.

## 3 Data and discussion of results

In 2014, China proposed the new energy-security strategy of “four revolutions and one cooperation,” highlighting that it should promote China's energy consumption, energy supply, energy technology, and energy system revolution and strengthen international cooperation on energy. Since 2015, the Chinese government has implemented a series of industrial policies to vigorously promote IESs, and the State Council, National Development and Reform Commission, National Energy Administration, SGCC, and other departments have issued several industrial policies related to IESs. Policy documents reflect government actions, which can truly reflect the intentions and purposes of the government's decision makers [37,38]. This study uses QAR methods based on grounded theory, with the help of NVivo and ATLAS software (through, e.g., the “word cloud” and “coding clustering” functions) to explore and extract policy information [46].

### 3.1 Policy sample selection

**Fig. 2:**
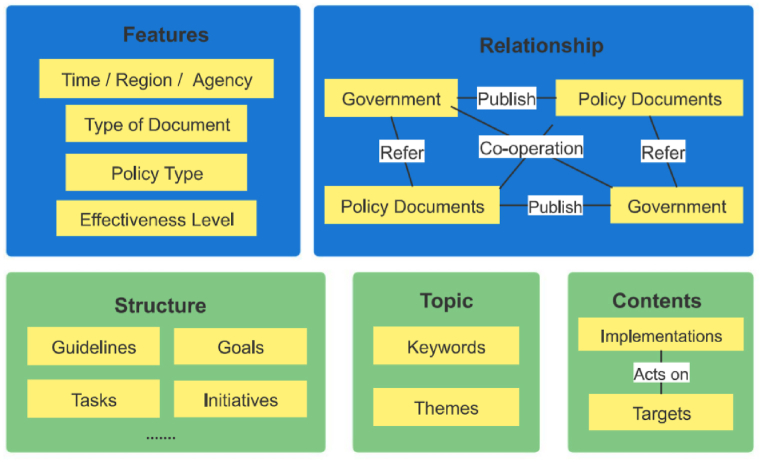
Policy analysis framework.

Fig. 2 reports that the policy analysis framework generally includes concepts on policy features, relationship, text structure, text topics, and contents. In this study, we selected the 2015–2022 publicly promulgated IESs policy documents as our research samples, and key contents of policy texts are derived through the word frequency analysis function of the qualitative analysis software. This is based on the word frequency results from the word cloud analysis, the contents of all sample policies are coded, reference points are summarized, and important text concepts are clustered. Accordingly, we analyzed the focus of the policies related to IESs and propose corresponding countermeasures to respond to shortcomings and deficiencies.

Texts of IESs policies selected in this study were all sourced from public data, mainly through search keywords “integrated energy services” and “energy internet.” Excluding departmental regulations and informal decision-making documents, a group of 21 policy documents between 2015 and 2022 was compiled as a sample for the study. Table 1 demonstrates the key elements of the sample policy.

**Table 1:** Key elements of China's industrial policy related to IESs in recent years.

Year	Key elements of the sample policy
2022	Promoting the high-quality development of new energy in the new era
2021	Promoting the integration of power source, electricity grid, energy load, energy storage, and multienergy complementary
2020	Expanding investment in strategic emerging industries
2019	Promoting the development of IESs business (2019–2020) through the National Grid Action Plan
2018	Requesting the establishment of the technical committee on the standardization of IESs in the energy industry the three-year action plan to win the Blue Sky Defense War
2017	The first batch of multienergy complementary integration and optimization demonstration projects opinion of the state grid corporation on the development of IESs business in provincial companies
2016	Launching the Internet Plus Smart Energy Demonstration Projects
2015	Launching the Internet Plus action plan

*China's Energy Ambitions (Four Revolutions and One Cooperation): Energy Consumption Revolution, Energy Supply Revolution, Energy Technology Revolution, Energy System Revolution, and Strengthen International Cooperation on Energy in all aspects.

### 3.2 Main results analysis: high frequency words and keyword clusters

#### 3.2.1 Policy text analysis of the sample based on word cloud and word frequency

In these 21 sample policy documents, irrelevant parts of the IESs were eliminated to create filtered policy texts. Filtered policy texts were then imported through NVivo and ATLAS (using the “word frequency query” function), text content language was set to “Chinese,” running condition was as “synonyms” and “with a minimum length of 2,” and invalid keywords were added to the “list of deactivated words,” finally generating a policy word cloud of IESs. Table 2 reports that through the statistics and analysis of high frequency words, sample policy documents cover the full period of China's 13th Five-Year Plan and early part of the 14th Five-Year Plan. Moreover, most frequent policy documents are obviously energy-related, and the whole policy documents of IESs cover a wide range of aspects, wherein keywords such as Projects, Management, Construction, System, Environment, Standards, and Demonstration appear more frequently.

**Table 2:** Statistics of high frequency words in policy samples on IESs.

Stage	High frequency words (including synonyms) and number of times
China's 13th Five-Year Plan (2015–2020)	Projects (785), Management (776), Regions (654), Construction (624), Services (519), System (467), Environment (404), Standards (398), Demonstration (330), Enterprises (316), Technology (310), Facilities (300), Clean (293), Internet (270), Electricity (235), Heating (232) Electricity Grid (230)
China's 14th Five-Year Plan (2021–2022)	Green (314), System (262), Project (241), Power (198), Construction (174), Enhancement (172), Management (164), Trading (151), Mechanism (149), Improvement (145), Support (139), Promotion (137), Clean (136), Enterprise (125), Standards (120), Technology (107), Transformation (103), market (87), resources (82), security (81), innovation (80)

**Fig. 3:**
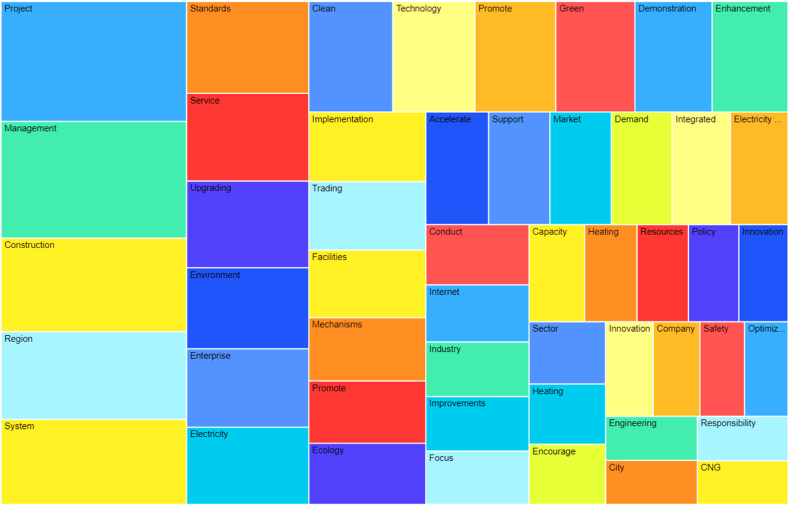
Word cloud of the policy samples on IESs.

In Fig. 3, the larger the area of the word box in the figure, the more frequently it appears in the policy document, and the higher the degree of government attention. Based on the statistics of word frequency and analysis word cloud, during the formulation of policy, the focus of attention is mainly concentrated on project construction, management system, clean and green, and energy facilities.

#### 3.2.2 Policy text analysis of the sample based on key phrases

We can initially explore the government's tendency and purpose in formulating IESs policy using qualitative analysis software to do word frequency analysis. However, this can only be used as a visual reference; word frequency analysis splits text phrases into fragments, which is not accurate enough for deeper analysis and research. Therefore, by analyzing specific key phrases, the semantic and contextual accuracy of the sample policy documents can be enhanced. By setting “integrated energy” or “integrated energy services” as key phrases to analyze the text of the sample policy documents and eliminate inappropriate results, through textual concept clustering of key phrases, we find that China's policies on IESs have initially formed seven categories, covering the industrial life cycle of IESs from Planning, Construction, Promotion, Management, Operation, Criteria, and Vision (Table 3).

**Table 3:** Key phrase clustering on the text of the IESs policies.

Category	Key phrase clusters
Planning	Enhance IESs planning and design in all aspects
	Emission reduction effect based on IESs
	Integrated energy planning and consulting
	Planning and developing of emerging businesses such as third-party IESs
Construction	All the units should strengthen the construction of IESs demonstration projects
	Building multienergy synergistic integrated energy micronetworks
	Constructing a new intelligent IESs system
	Innovate the construction management system of IESs projects
	Promote the construction of integrated energy network infrastructure
Promotion	Create a propaganda platform for the company's IESs
	Develop a comprehensive energy operation information infrastructure
	Information-interactive IESs platform
	Strengthen the information and operation management of IESs
Management	Establish IESs companies
	Expand the influence of the company's IESs brand
	Expand the payroll of provincial-level IESs companies
	Forming an IESs ecosystem
	Form the alliance of IESs industry
	Provincial electricity grid companies to conduct IESs business
	Support provincial-level IESs companies
Operation	Forming the conception of IESs
	Human resources of IESs companies
	Proactively conduct IESs business
	Provincial electricity grid companies to conduct IESs business
	Strengthen and expand the IESs business
Criteria	Formulate project management methods for IESs companies
	Improve procurement of materials for province-level IESs companies
	Provide intelligent IESs
	Standardization of IESs
	Standardization technology of IESs in the energy industry
Vision	Cooperate to expand the market of IESs
	Customize IESs holistic solutions
	Establish a bank of IESs projects
	Establish a mechanism for regional IESs
	Establish a special award for IESs
	Maximize usage of large-scale integrated energy bases
	Provide diversified IESs
	Provide flexible and intelligent integrated energy supply and value-added services
	Transformation of electricity suppliers to IESs providers

For implementing IESs, China has exerted its best efforts to alleviate externalities and market failures by maximizing government intervention and industrial policy incentives. Using “energy structure” as the key phrase to analyze the text, the analysis was extended to adjacent areas of sample policy documents’ material to obtain long sentences and paragraphs. Eight reference points were then obtained from the sample, and those with similar content were excluded and six retained reference points were analyzed. Hence, IESs implementation in China has developed a clear policy implementation path, from the “background analysis” to “policy targets.”

i) *Background analysis.* Rapid change in global climate calls for a more sustainable path for China's energy system. IESs serves as a novel solution for meeting the diversified energy production and consumption of end-customers, through energy planning and design, engineering investment and construction, multienergy operation services, and investment and financing services.
ii) *Environmental priorities.* Using economy, legal, technical and necessary administrative order, adjust and optimize energy, industry, and transportation structures; strengthen regional joint prevention and control; and continue preventing and controlling air pollution.
iii) *Direction of guidance.* To accelerate adjustment of energy structure, promote the construction of a clean and low-carbon energy supply system, highlighting the basic consensus and orientation of energy structure to nonfossil energy transformation.
iv) *Framework of measures.* Strengthen scientific and technological innovation in the field of energy, promote transformation of the development mode of the power industry and optimization of the energy structure, improve the quality and efficiency of the energy industry, and increase the proportion of renewable energy generation and distributed energy system generation in the power supply.
v) *Customer groups.* Conduct a census of energy structure information of high-voltage customers, aim to improve customers' energy efficiency and reduce energy costs, implement changes in traditional energy-saving methods by applying the ubiquitous Internet of Things (IoTs) technology, and realize system- and platform-level comprehensive energy efficiency improvement.
vi) *Policy targets.* By 2025, the energy structure is significantly optimized, allocation of energy resources is more reasonable, utilization efficiency is significantly improved, level of clean production is continuously improved, total emissions of major pollutants are continuously reduced, intensity of carbon emissions is significantly reduced, ecological environment is continuously improved, and production, circulation, and consumption systems of green low-carbon cycle development are initially formed.

From the qualitative analysis of the sample policy documents, the attitude of the Chinese government and large energy monopoly of state-owned enterprises toward IESs is positive in terms of encouragement and support. In 2020, IESs became one of China's national strategic emerging industries. Considering the policy content over the years, initially, China encouraged specialized energy service companies to cooperate with customers to conduct “Internet + Smart Energy” using the model of Energy Management Contract (EMC) [1]. Subsequently, China emphasizes that the proportion of new industrial parks adopting integrated terminal functions has reached about 50 % and that of existing industrial parks implementing integrated energy tertiary utilization transformation has reached 30 %. Moreover, China has increased the construction of microgrids, encouraged industrial parks to promote independent green energy balancing transactions, and conducted pilot distributed power supply direct load supply.

No matter how industrial policy is constrained and guided, enterprises engaging in IESs business need to carefully consider the policy environment, economic level, energy endowment, energy load, energy structure, and other factors [2,47]. China's market exploration strategy for IESs implementation is to build demonstration projects based on information systems, with high viscosity services as the core and win–win cooperation. In industry business expansion, China's regional power enterprises establish IESs companies that focus on various industrial parks, industrial enterprises, public buildings, residential buildings, new energy generation and other areas and drive industry business expansion by replicating demonstration projects. In terms of regional business expansion, most enterprises are monopolized by state energy enterprises, which promote IESs in areas with high and concentrated energy consumption loads, industrial energy dominance, and strong policy support.

### 3.3 Discussion and analysis

Overall, IESs development in China remains in its infancy. China is currently promoting the IESs market through various energy companies, mainly those held by state-owned capital, in high-load energy-using areas, high-quality industrial parks with concentrated industries, and policy-friendly areas. This maximizes favorable policies and incentives to compensate for the high costs in the early stages of new business development.

#### 3.3.1 Considerable heterogeneity in energy consumption across China

In 2021, China's annual energy consumption will total 5.24 billion tons of standard coal, constituting an increase of 5.2 % over the previous year. Coal consumption increased by 4.6 %, constituting 56 % of the total energy consumption, which is down 0.9 % from the previous year. As IESs mainly engage in electrical energy, we use the distribution of electrical load as a reference basis, combined with the consumption of natural gas and other energy sources for comprehensive consideration.

**Fig. 4:**
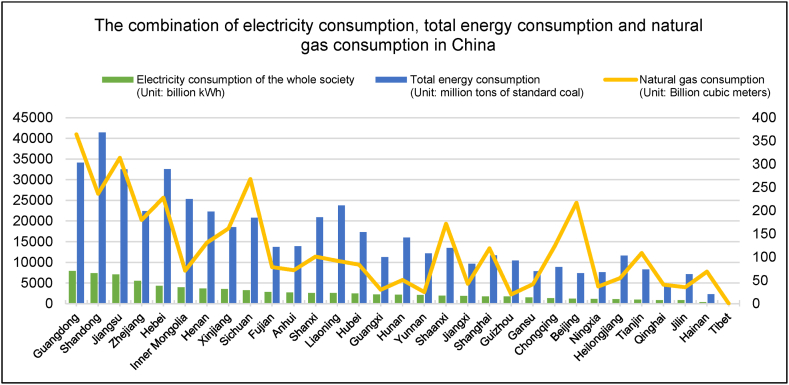
Combination chart of electricity, total energy consumption, and natural gas in China (2021).

According to the China Energy Statistics Yearbook (2021), electricity consumption in Guangdong reached 632.3 billion kWh, and Jiangsu is the second region in China to exceed 600 billion kWh of annual electricity consumption (Fig. 4). Meanwhile, Shandong, Zhejiang, Henan, Hebei, and Inner Mongolia all consume more than 300 billion kWh of electricity, Guangdong, Jiangsu, and the five regions account for nearly 50 % of the country's total electricity consumption. Additionally, Sichuan and other six regions'(including Fujian, Liaoning, Shanxi, Xinjiang, Anhui, and Hubei) electricity consumption reach more than 200 billion kWh, and the 14 regions accounted for more than 70 % of the total amount of electricity. In terms of natural gas consumption, the top three natural gas consumers in China were Guangdong, Jiangsu, and Sichuan, reaching 36.4 billion, 31.37 billion, and 26.8 billion m^3^, respectively. In terms of heat load, the total centralized heating area in China is now over 10 billion m^2^. Heat supply use can be divided into industrial and commercial heat supply and residential heat supply, of which the former accounts for about 70 % of the total heat demand. No obvious seasonal or geographical differences between the South and North were confirmed, except those related to the industrial field of application. Compared with residential heat, industrial heat supply is less concentrated and is dominated by a more decentralized form of heat supply. Future development directions may aim to conduct centralized industrial heating according to local conditions.

Considerable heterogeneity in energy consumption can be found across Chinese regions, and choosing a more appropriate method to implement effective energy saving and emission reduction is particularly important, the IESs is one of the energy transformation measures that China is currently vigorously developing.

#### 3.3.2 IESs industrial policies promote parks development

Almost half a century has passed since the Chinese economic reform, which coincided with the construction of China's industrial parks and special economic zones in four coastal cities: Shenzhen, Zhuhai, Shantou, and Xiamen. China's various industrial parks are key in the country's reform and opening-up strategy, and their development process coincides with China's openness to the outside world [7].

Currently, China has more than 350 national-level industrial parks, more than 1200 provincial-level industrial parks, more than 1000 large-scale urban-level industrial parks, and tens of thousands of industrial parks below the county-level, which contribute more than 30 % to China's economy. Common types of industrial parks include port logistics parks, science and technology innovation parks, industrial-city complexes, business office parks, and tourism resorts. Among them, almost 70 % of China's energy consumption is concentrated in various industrial parks, contributing almost 30 % of total carbon emissions in the country. Additionally, the large energy demand of customers in industrial parks facilitates economies of scale by reducing costs, leading to greater likelihood of forming diversified energy value-added services. Therefore, various types of parks, especially high-tech manufacturing industrial parks, are the most critical and appropriate market for the IESs [48,49].

**Fig. 5:**
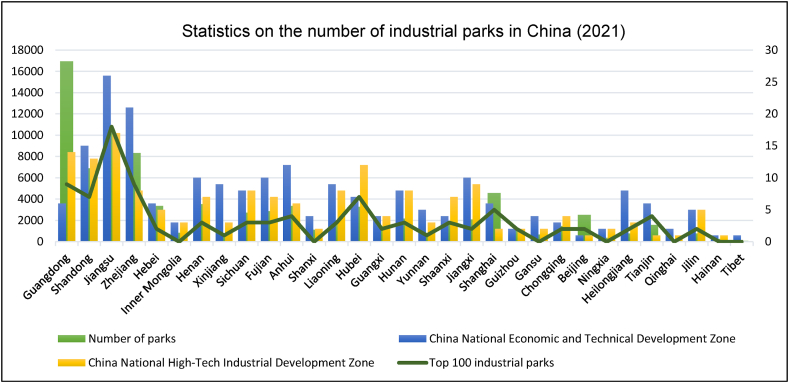
Statistics of industrial parks in China (2021).

By referring to the China Industrial Park Sustainable Development Blue Book (2018) published by Tongji University, the top ten industrial parks according to their sustainable development capacity are Beijing Zhongguancun, Shanghai Zhangjiang, Suzhou Industrial Zone, Guangzhou Economic Development Zone, Wuhan East Lake, Chengdu High-tech Zone, Beijing Economic Development Zone, Hefei High-tech Zone, Shenzhen High-tech Zone, and Tianjin Economic Development Zone (Fig. 5). In terms of the number of top 100 industrial parks selected by Chinese provinces, Jiangsu tops the list with twenty industrial parks, followed by Shandong with eleven industrial parks. In third place is Guangdong with nine industrial parks, fourth place is Zhejiang with seven, and tied for fifth place are Anhui and Hunan with four industrial parks.

Owing to the wide geographical distribution and uneven development level of these parks, and lack of economies of scale in IESs, the incentive mechanism of industrial policy should be maximized. Moreover, the policy environment, economic level, energy endowment, energy load, energy structure, and other factors of each industrial park must be considered. Industrial parks with suitable conditions must be selected for the establishment demonstration projects to gradually realize economies of scale effect for IESs [48,50].

#### 3.3.3 IESs industrial policies drive achieving carbon neutrality goals

In November 2020, China proposed the country's goal to achieve “emission peak and carbon neutrality” and has since continuously introduced various policies and strategies to create a carbon neutral society [[51], [52], [53]]. We have clustered some public policies related to IESs and carbon neutrality in China into Table 4, and five main policy directions are found: Energy Conservation Monitoring, Clean Power, Investment Finance, Green Technology, and Carbon Market. China has formulated a “1 + N” policy system to achieve the carbon neutrality target. Our analysis supports that China is committed to promoting the IESs policy through saving energy and reducing carbon emissions. Implementing IESs, especially in industrial parks with concentrated energy consumption and high-carbon emissions, can help China achieve carbon neutrality.

**Table 4:** China's public policies related to IESs and carbon neutrality (as of January 2022).

Policy Orientation	Policy Name
Energy Conservation Monitoring	Energy Efficiency Benchmarking Levels In Key Areas of High Energy-Consuming Industries (2021)
	Several opinions on strict energy efficiency constraints to promote energy saving and carbon reduction in key areas
	Notice on further strengthening the energy-saving monitoring work
Clean Power	Notice on the national coal power unit transformation and upgrading
	Guidance on accelerating the development of new energy storage
	Guidance on promoting the integration of power, network, load, storage and multienergy complementary development
Investment Finance	Number of views on further promoting the reform of the investment project approval system
	Notice of the pilot work on climate investment and financing
	Opinions on encouraging and supporting social capital to participate in ecological protection and repair of the heavy
Green Technology	Notice on the issuance of the Action Plan for Carbon Neutral Science and Technology Innovation in Higher Education Institutions
	Notice on the issuance of the “National High-tech Zone Green Development Special Action Implementation Plan”
	Notice on the issuance of “green technology promotion catalog” (2020)
Carbon Market	Guidance on accelerating the construction of a unified national electricity market system
	Notice on the supervision and management of the quality of convergence in the national carbon emission trading market
	About the Release of the Carbon Emissions Registration Management Rules (for Trial Implementation) and the Carbon Emissions Trading Management Rules (for Trial Implementation)
	Announcement of “Carbon Emissions Settlement Management Rules” (for Trial Implementation)

#### 3.3.4 Provide targeted services for typical customers

Currently, China's IESs mainly takes the electricity power system as the core, which can achieve coupling and coordination between various energy sources. However, enterprises have their own independent industrial chains in conducting the business of power supply, heating, cooling, water supply, and gas supply. However, different energy subsystems remain independent in planning, design, construction, operation, and management, and serious information barriers exist between them [47,49]. IESs, being a relatively new process of energy system reconstruction, should be implemented by gradually dismantling barriers between different energy industries and energy companies.

Through the niche market, energy consumption characteristics, and necessary targeted services, we find that the IESs aim to establish a unified energy market and management system, promote interconnection of various energy infrastructures, and realize the coordinated and efficient utilization of multiple energy sources. According to the actual situation of customers and targeted services that IESs should provide, mainly focusing on their energy consumption characteristics, we classify the typical customers (niche markets) into the following four categories.

i) *Enterprises in the industrial parks.* This includes various manufacturing, process, and R&D industrial parks. The energy consumption characteristics of such customers include high energy prices, high energy demand and energy use in various forms, which are mainly distributed in steel, building materials, chemical and nonferrous industries. These customers should be targeted with services including integrated electricity, cooling, and heat supply are the necessary targeted services. To realize the multienergy synergy supply, comprehensive gradient utilization and low-grade waste heat utilization are key. Providing industrial enterprises with energy efficiency diagnosis, waste heat recovery, motor energy saving, green lighting, and equipment generation operation and maintenance are necessary. Finally, electric energy substitution and energy efficiency improvement services should be conducted.
ii) *Large public buildings.* This includes government offices, schools, hospitals, intelligent buildings. The energy consumption characteristics of such customers include high-density sectors of building energy consumption with considerable energy-saving potential. These customers should be targeted with services including integrated energy supply of electricity, heat and cooling for large public buildings. Energy-saving capabilities of high-voltage alternating current, lighting, and power should be promoted to improve energy use efficiency of buildings. Other services, such as optimal control and energy trustee, are also available.
iii) *Data centers.* This includes energy internet companies and banks. The energy consumption characteristics of such customers include high energy consumption, especially high cooling demand. These customers should be targeted with services that help meet their energy demand, such as electricity and cooling, energy consumption monitoring and optimization. Energy-saving solutions that improve energy efficiency are key.
iv) *Densely populated residential areas.* This includes home centralized heating and smart-homes. The energy consumption characteristics of such customers include strong demand for centralized heating. For such customers, the market potential for clean heating and cooling is enormous, and cooperation with real estate developers can be strengthened to provide centralized heating and cooling supply services for residential buildings. Additionally, the promotion of smart communities and smart-homes to improve the level of electrification of residents also has broad market development prospects.

## 4 Policy implications

By implementing a QAR methodology and analyzing 21 sample policy documents related to IESs, this study reports that IESs in China will synchronize with the construction of urban governance systems and gradually evolve into urban-level energy information platforms. In this process, a considerable number of innovative enterprises will be created to promote the growth of regional IESs ecosystem. Simultaneously, a considerable number of regional IESs will be created, specialized platforms for energy IoTs will be born, and novel IESs models will emerge [18,54].

Existing demonstration projects or engineering cases demonstrate that IESs implementation in China's industrial parks is currently dominated by large energy enterprises (e.g., SGCC), and most of which are state-owned capital backgrounds. The “Action Plan on Promoting the Development of Integrated Energy Services Business 2019–2020” and the “Integrated Energy Services Market Strategy Study and Recommendations” issued by SGCC report that the key businesses of IESs mainly include energy supply construction, energy-saving services, clean energy and electric energy replacement, integrated energy operation and maintenance, energy purchase, sale, and financial services.

Industrial parks and large public buildings (including government offices, schools, hospitals, hotels, shopping malls, etc.) mainly use electricity in their energy consumption, but individual energy consumption levels vary greatly. These target customers generally have high energy consumption levels, small load fluctuations, and rapid energy demand growth. IESs can then be used to strengthen energy management. Although the project's ROI is not initially high, the income becomes stable in later stages and the risks become more controllable, which has good demonstration and promotion significance [55,56].

The above analysis and discussion are only a qualitative study of the industrial policy and actual performance of China's IESs implementation. However, the policy text analysis cannot conduct deeper research on the theory and problems behind the text phenomenon. The primary objective of this QAR study is to reveal the policy text and governmental inclinations towards IESs. However, the vast amount of technical explanations and descriptions present in the sample documents indicate that IESs are being extensively studied from various perspectives across multiple research areas. To effectively address negative externalities and market failures associated with IESs and expedite the energy transition through policy guidance and government intervention, future studies should follow the direction indicated by policy texts and concentrate on the following research topics.

i) Develop engineering research on IESs in according to policy guidance. IESs engineering technology research, mainly including distributed energy supply, energy conversion and storage, multienergy flow Supervisory Control And Data Acquisition and security control, energy big data and artificial intelligence, risk management, investment and transaction portfolio optimization. Development constraints of IESs, mainly including the game theoretical perspectives among energy enterprises, integrated operation and maintenance service, EMC, electric energy replacement service, multienergy retail, energy agency trading, etc.
ii) Fully utilize the policy to carry out business model innovation of IESs. IESs business model research, mainly including internal benefit research, external benefit research. Currently, the research on the business model of IESs, especially the business model of IESs in industrial parks, is relatively weak. IESs has a wide range of internal revenue sources, including energy sales, agency transactions, design consulting, equipment leasing, information services, energy-saving sharing, and other services. The external benefits of IESs are unique to industrial parks, by implementing IESs in industrial parks, using smart energy management, multienergy retail and other technical and economic measures and relying on internal and external energy exchanges to obtain value-added benefits, these business models and economic behaviors are worthy of in-depth research.

## 5 Conclusion

Currently, the key to IESs implementation is coordination and optimization. However, this remains limited to the energy industry itself. Although energy is the key to economic and social development, it should gradually deconstruct the barriers of different industries in the future, organic integration with other industries to achieve a full range of expansion, and to realize “IESs + manufacturing,” “IESs + transportation,” “IESs + agriculture” and other kinds of industrial synergy development system, these innovative industries are the critical areas for future research on IESs.

In this study, we adopted the QAR method based on grounded theory and used NVivo and ATLAS software to analyze the text by examining high-frequency words and keyword clusters. Although our approach differs from previous studies that have adopted engineering-based perspectives, our study is not without limitations. For instance, we recognize that our sample size is small, which may affect the generalizability of our findings. Moreover, it may be difficult to generalize our findings to the population at large, and our ability to accurately infer the government's intentions and purposes may also be limited. Additionally, the subjective nature of the analysis process may have influenced our ability to summarize the information effectively. Despite these limitations, our study provides a unique perspective on understanding IESs using the QAR method, which can help advance the field and guide future research.

From the perspective of China's IESs implementation, countries worldwide must deconstruct the barriers between energy industries and improve the system of energy management in the process of developing IESs. Additionally, it is necessary to establish a fair competition, open and orderly energy market trading system, improve the energy market access system, and encourage various enterprises to actively participate in energy and derivatives market transactions. Governments should also proactively participate or intervene by policies to maximize market vitality and reduce the externalities, realize the economies of scale as soon as possible, and gradually realize the good vision and good effects of implementing the IESs industrial policy.

## Ethics declarations

All participants provided informed consent to participate in the study.

## Data availability statement

The data samples used in this study are publicly collected and organized. Researchers and interested parties may request access to these data samples by sending an email to mr.zhangcan@ucass.edu.cn.

## Ethics committee approval

This research did not involve any ethical risks or human subjects. Consequently, no ethics committee approval was required. All interviews, surveys, and questionnaires were conducted in accordance with established ethical guidelines, and informed consent was obtained from all participants.

## CRediT authorship contribution statement

**Shengzhong Huang:** Supervision, Project administration, Funding acquisition. **Can Zhang:** Writing – review & editing, Writing – original draft, Visualization, Validation, Software, Resources, Methodology, Investigation, Formal analysis, Data curation, Conceptualization. **Wei Li:** Validation. **Mengkai Liu:** Visualization.

## Declaration of competing interest

The authors declare that they have no known competing financial interests or personal relationships that could have appeared to influence the work reported in this paper.

## List of abbreviations

IESs: Integrated energy services
QAR: Qualitative Analysis Research
SGCC: State Grid Corporation of China
EU: European Union
US: United States of America
EMC: Energy Management Contract
IoTs: Internet of Things
ROI: Return On Investment
